# Molecular Alterations and Severe Abnormalities in Spermatozoa of Young Men Living in the “Valley of Sacco River” (Latium, Italy): A Preliminary Study

**DOI:** 10.3390/ijerph191711023

**Published:** 2022-09-03

**Authors:** Pasquale Perrone, Gennaro Lettieri, Carmela Marinaro, Valentina Longo, Simonetta Capone, Angiola Forleo, Sebastiana Pappalardo, Luigi Montano, Marina Piscopo

**Affiliations:** 1 Department of Biology, University of Naples Federico II, Via Cinthia, 21, 80126 Naples, Italy; 2 Department of Precision Medicine, School of Medicine, University of Campania “Luigi Vanvitelli”, 80138 Naples, Italy; 3 Institute for Microelectronics and Microsystems (IMM), National Research Council of Italy (CNR), 73100 Lecce, Italy; 4 Reproduction and Fertility Center, Via A. Vitozzi, 50, 00128 Rome, Italy; 5 Andrology Unit and Service of Lifestyle Medicine in UroAndrology, Local Health Authority (ASL) Salerno, Coordination Unit of the Network for Environmental and Reproductive Health (EcoFoodFertility Project), Oliveto Citra Hospital, 84020 Oliveto Citra, Italy; 6 PhD Program in Evolutionary Biology and Ecology, University of Rome Tor Vergata, 00133 Rome, Italy

**Keywords:** sperm nuclear basic proteins, human protamines, human spermatozoa, male fertility, spermatozoa morphology, volatile organic compounds, reproduction, protein–DNA binding

## Abstract

The Valley of Sacco River (VSR) (Latium, Italy) is an area with large-scale industrial chemical production that has led over time to significant contamination of soil and groundwater with various industrial pollutants, such as organic pesticides, dioxins, organic solvents, heavy metals, and particularly, volatile organic compounds (VOCs). In the present study, we investigated the potential impact of VOCs on the spermatozoa of healthy young males living in the VSR, given the prevalent presence of several VOCs in the semen of these individuals. To accomplish this, spermiograms were conducted followed by molecular analyses to assess the content of sperm nuclear basic proteins (SNBPs) in addition to the protamine-histone ratio and DNA binding of these proteins. We found drastic alterations in the spermatozoa of these young males living in the VSR. Alterations were seen in sperm morphology, sperm motility, sperm count, and protamine/histone ratios, and included significant reductions in SNBP–DNA binding capacity. Our results provide preliminary indications of a possible correlation between the observed alterations and the presence of specific VOCs.

## 1. Introduction

### 1.1. Preface

In Italy, there are some areas that have high levels of pollution. One region with substantial pollution is Italy’s Land of Fires, an area between Naples and Caserta that has been overrun by illegal waste dumping and toxic fires, prompting the establishment of the EcoFoodFertility project, which is a multicenter biomonitoring study with the aim of developing a better understanding of the environmental impact of toxicants on human health (https://www.ecofoodfertility.it/, accessed on 12 July 2022).

### 1.2. The Issue

This study forms part of the EcoFoodFertility project and focused on another district with significant pollution named the “Valley of Sacco River” (VSR) (Latium, Italy) [1,2,3]. The VSR is an area located mostly in the province of Frosinone and partly in the southern area of Rome. There is an important industrial district in the VSR. In fact, due to the intense industrial and especially chemical activity of the Colleferro plants, there has been an overload of pollutants in this area, which have contaminated the soil and groundwater over many years, creating problems throughout the food chain. The spills of illegal industrial waste have led the river to become a contaminated site; contaminated water has been used for irrigation purposes, with negative consequences on both animals and the population living in the area, and despite some reclamations, the site is under surveillance. Soil studies in the industrial area of this zone revealed the cumulative accumulation of different toxic substances and organic pesticides, especially through water and food [1], and very high levels of chromium and dioxins.

### 1.3. Pollution and Reproduction

In recent years, there has been a great deal of evidence that pollution has a strong effect on the reproductive health of living organisms; in fact, important effects have been demonstrated in spermatozoa [4,5,6,7,8,9,10,11,12], which are particularly sensitive to environmental changes [13,14,15,16]. It is well-known that various pollutants can cause an imbalance of reactive oxygen species (ROS) [5,7,17,18,19,20] and that the resulting oxidative stress events have the potential to induce alterations in sperm morphology, number, and motility [13,14,15,16]. In addition, these processes can cause oxidative damage to DNA [7]. Environmental pollution is also reported to alter disease susceptibility in different populations, and semen quality has proven to be a potential predictor of sensitivity to viral insults in highly polluted areas [21,22]. An early biomarker of environmental exposure, such as Kallikrein-related serine peptidase 3, was also recently identified in young women [23]. Moreover, volatilome fingerprinting of human biofluids by both chemical analytical techniques and gas sensor systems is attracting interest in human biomonitoring as a new approach to explore the internal chemical environment due to multiple exposomic stressors and discover potential VOC biomarkers useful in pollution health impact assessment [24]. Much research has been done in recent years because the problem of infertility due to environmental pollution is thought to be increasingly common [25]. Recently, it was also shown that reduced male fertility and gonadal development, as well as cancers of the reproductive system due to exposure to organic and inorganic pollutants, can be counteracted by flavonoids [26]. Spermatozoa are highly reactive to the pro-oxidant effects of environmental pollutants essentially for two reasons: as a result of the reduced volume of cytoplasmic space, with less antioxidant defense [27], and because sperm membrane lipids, especially rich in polyunsaturated fatty acids, are the target of reactive oxygen species (ROS) [28]. This is why spermatozoa have been recognized as an “ideal” biomarker of environmental pollution and early sentinels of human health [13]. In human spermatozoa, there are two types of SNBPs to package the genome in the highly condensed sperm nucleus: protamines (P1 and P2) and histones [29,30]. The canonical ratio between protamines and histones is about 85%:15%, and it appears essential to maintain this ratio between these two types of proteins for male fertility [31].

In a very recent work, it was demonstrated that several volatile organic compounds (VOCs) are present at high levels in the semen of young men living in the VSR [32]. VOCs are a heterogeneous category of substances that includes organic chemicals that have a high vapor pressure at room temperature. Taken together, they exhibit high volatility but show extremely different physical and chemical behaviors. VOCs are also emitted from solids or liquids, so exposure to airborne VOCs is unavoidable. Most VOCs are not acutely toxic but may have short- and long-term adverse health effects. Specifically, in the VSR area, considering the pervasive chemical exposure of the population caused by environmental pollution, exposure to VOCs via multiple exposure sources (mainly air, water, and food) has occurred with hidden dangerous cumulative effects on health.

### 1.4. The Aim of Work

Therefore, in the present work, we investigated whether these pollutants could produce alterations in the spermiogram parameters and SNBP properties of young men in the VSR. To this aim, we performed molecular analyses to assess the protamine/histone ratio and the DNA binding of these proteins. Finally, we analyzed the morphology of the spermatozoa of young men in the VSR and tried to find a possible correlation between morphological, molecular, and other seminal changes and the presence of specific VOCs.

## 2. Materials and Methods

### 2.1. Reagents

All reagents used for this work were of analytical grade and were purchased from Sigma-Aldrich (Merck KGaA, Darmstadt, Germany).

### 2.2. Ethical Statements

All methods were carried out in accordance with the Code of Ethics of the World Medical Association (Declaration of Helsinki) guidelines and regulations. All experimental protocols were approved by the Ethical Committee of the Local Health Authority Campania Sud-Salerno (Committee code n. 43 of 30 June 2015). Informed consent was obtained from recruited subjects before sample collection.

### 2.3. Recruitment

**Figure 1:**
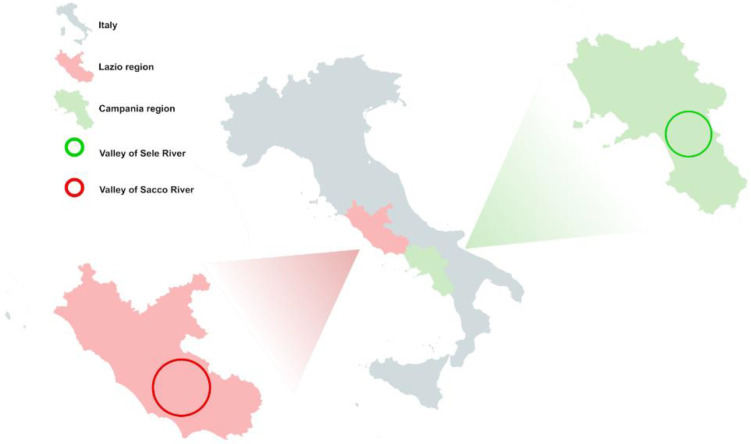
Map of the geographical areas selected for the recruitment. The green circle indicates the Valley of Sele River in the Campania region; the red circle indicates the Valley of Sacco River (VSR) area in the Lazio region. The green and red circles indicate the geographical position of the two areas in the respective regions.

Subject recruitment was conducted between October 2017 and November 2018, as part of a pilot study of the EcoFoodFertility project (www.ecofoodfertility.it, accessed on 12 July 2022). The semen samples comprising the “pollution exposed” group came from individuals living in the VSR area, a region that extends principally in the province of Frosinone and to the south of Rome. The geographical areas considered in our study (Figure 1) differ considerably in the concentrations of various pollutants present. The Valley of Sele River (Control group) is a low environmental impact area (https://www.arpacampania.it/, accessed on 12 July 2022). This area has no known illegal disposal of toxic waste and has an economy based primarily on low- to medium-scale agriculture. The Valley of Sele River, based on the concentrations of pollutants detected in the area, falls into the ‘maintenance’ category and is included in the lower diffuse emission zones for sulfur oxide and medium diffuse emission zones for nitrogen oxide, carbon monoxide, volatile organic compounds, and suspended particles, so the risk thresholds for human health are not exceeded [33]. The VSR is an area where the concentrations of various pollutants, including heavy metals and pesticides, are much higher than the national averages [32].

The chemical analyses carried out ascertained wide-ranging environmental pollution linked to the contamination of the Sacco River by toxic waste dumps of industrial origin to which animals of zootechnical interest and the human population have been exposed [1]. Control semen samples used for comparison were obtained from the San Francesco d’Assisi Hospital of Oliveto Citra-Province of Salerno, a municipality belonging to a low environmental impact area called the Valley of Sele River. The selected participants were healthy males aged between 18 and 30 years and residents of at least 10 years in the areas under study with no known chronic diseases (diabetes or other systemic diseases), no varicocele, and no factors that could affect sperm quality (such as fever, medications, exposure to X-rays, etc.). Subjects were also non-drinkers and non-smokers with no reported history of drug abuse and no occupational exposure.

Data were collected by questionnaire and physical examination, including urogenital assessment (testis volume and transrectal prostate assessment). At the time of enrollment, a code number (VSR1, VSR2, VSR3, …, VSRn) was assigned to each volunteer by the recruiting andrologist (the recruiter) to maintain privacy. Each code number was uploaded to a computer database along with personal information and clinical information. A total of 80 samples were analyzed from the “low pollution” area and 76 samples from the “high pollution” VSR group.

### 2.4. Sample Collection

Human semen samples were collected upon morning masturbation after 3–4 days of sexual abstinence in sterile containers. Semen samples underwent routine tests to evaluate sperm quality (semen volume, sperm concentration, motility, and morphology) according to the World Health Organization (WHO) guidelines (World Health Organization, 2010) [34]. After incubation to 37 °C for 30–40 min and complete liquefaction, the sperm analysis was performed according to WHO [35], using both the classical microscope for optical evaluation with Makler’s chamber and one automated sperm analysis using the Lenshooke Semen X1 Pro system (Bonraybio Co., Ltd., Dali Dist., Taichung City, Taiwan), an innovative automatic analytical certified CE IVD system, equipped with microscope-integrated optics and an artificial intelligence system. The following parameters were measured: sample volume, pH, sperm concentration, total and progressive motility, sperm morphology, and concentration of round cells.

### 2.5. SNBP Extraction from Spermatozoa

The semen was divided into 500 µL volume aliquots in 1.5 mL tubes. Semen samples were centrifuged for 30 min at 5500× *g* at 4 °C in order to purify spermatozoa. To extract protamines from spermatozoa pellets, the protocol described by Lettieri et al., 2020 [7] was used [7]. In brief, the spermatozoa pellet was resuspended in 500 μL of 1 mM Phenyl Methyl Sulfonyl Fluoride (PMSF). Thereafter, the sample was centrifuged at 4 °C for 10 min at 10,480× *g*; the obtained pellet was washed again with 1 mM PMSF and recentrifuged as described before. A total of 50 µL of 1 mM PMSF, 50 μL of 8 M guanidine hydrochloride, and 200 mM dithiothreitol (DTT) were added to the pellet, containing DNA and protein. The sample was incubated at room temperature for 30 min, and then 5 volumes of cold ethanol were added to the sample. The sample, after being at −20 °C for 1 h, was centrifuged at 4 °C for 15 min at 13,680× *g*, and the formed pellet, containing the proteins, was resuspended with 500 μL of 0.5 M HCl and incubated in the thermoblock at 37 °C for 5 min. Thereafter, the sample was centrifuged at 1000× *g* for 10 min at 4 °C, and the obtained supernatant, containing the sperm nuclear basic proteins, was precipitated with 20% Tricloro Acetic Acid (TCA) after incubation on ice for 1 h. SNBPs were obtained in the form of a pellet after centrifugation of the sample at 4 °C for 10 min at 14,000× *g.* The pellet was washed with 500 μL of a solution containing 1% β-mercaptoheptanol and 100% acetone and then recentrifuged for 10 min at 14,000× *g* at 4 °C. The final pellet was resuspended in about 50 µL of H_2_O and stored at −20 °C.

### 2.6. Electrophoretic Analysis of SNBPs

Acetic acid-urea gel electrophoresis (AU-PAGE) was used to analyze the SNBPs (i.e., histones and protamines) extracted from spermatozoa. AU-PAGE was performed as previously described in Lettieri et al., 2021 [36] using 9.0% (*w/v*) acrylamide (acrylamide:bisacrylamide 25:0.67). The gel, with a final volume of 8 mL, consisted of acrylamide/*N*,*N′*-Methylene-bis-acrylamide, 8 M urea, 5% acetic acid, 100 μL TEMED, and 140 μL of 10% APS. After gel polymerization, a pre-run of approximately 1 h was performed at a constant voltage of 150 V by using 5% acetic acid as a running buffer. For the pre-run, in each well, 20 μL of a solution containing 8 M urea and 5% acetic acid was loaded. After this step, a solution containing 4 μg of protein and 20 μL of a solution containing 12.8 M β-mercaptoethanol and 8 M molar urea were loaded onto the gel, and finally, after one hour, 2 μL of 100% acetic acid and 2 μL of 0.001% pyronin were added. The electrophoresis was performed at 100 V for about 1 h. After the electrophoresis, gels were stained with Coumassie Blue Brilliant R-250 as previously described [37]. The image was acquired using GelDoc Biorad, and densitometric analysis was performed using ImageJ ver. 1.53k software (https://imagej.nih.gov/ij/, accessed on 12 July 2022) supported by the National Institute of Health (NIH) (Rockville Pike, Bethesda, MD, USA). We classified the analyzed samples in the two areas according to the type of bands identified on AU-PAGE. In particular, the band at higher mobility represented the protamines P1 and P2, while the bands at lower mobility were the histones and other basic proteins. We defined the protamines/histones ratios by the densitometric analyses of the bands obtained in the gels. When there was a prevalence of histones and other basic proteins over protamines, we classified the sample as only-H, as shown in the majority of the analyzed samples. When there was a prevalence of protamines over histones and other basic proteins, the sample was classified as nCP/Hr, as shown in the majority of the analyzed samples.

### 2.7. Plasmid DNA Preparation

pGEM3 plasmid (2867 bp) from *Escherichia coli* HB 101 cells was prepared by using the HiPura Endotoxin free Plasmid DNA Midiprep Kit (HiMedia Laboratories Pvt. Ltd., Mumbai, India) following the precautions described in Carbone et al., 2012 [38], with the aim to achieve high amounts of super-coiled pDNA. After extraction of plasmid DNA, its concentration was evaluated by Spectrophotometer Nanodrop 1000 (Thermo Fisher Scientific, Waltham, MA, USA), and the plasmid DNA topological state was verified by gel electrophoresis of 1% agarose gels in 89 mM Tris-HCl pH 8.0, 2 mM EDTA, and 89 mM Boric Acid (TBE). For the electrophoretic mobility shift assay (EMSA) and DNA breakages/protection assays, the circular form of the plasmid was used.

### 2.8. Analysis of SNBP/DNA Binding

The analysis of SNBP/DNA binding was performed by EMSA following the procedure described in Lettieri et al., 2021 [11]. The assay was done on 1% agarose gel, in 1× TBE buffer. The samples were prepared as previously described [39] in distilled water containing a fixed amount of plasmid DNA (150 ng) and increasing amounts of SNBPs in order to obtain samples differing in the protein/DNA *w/w* ratio. The interaction between plasmid DNA and proteins was for 5 min at room temperature, and then TBE and the sample buffer were added to a final concentration of 1×. The electrophoresis was conducted at a constant voltage of 100 V for 30 min. At the end of the electrophoresis, DNA was visualized by means of ethidium bromide. Gels were acquired using a Gel-Doc system (BioRad, Hercules, CA, USA) through Image Lab 6.0.1 software (build 34; BioRad, Hercules, CA, USA). A densitometric analysis of the bands on the gel was performed using the software ImageJ ver 1.50 d [40].

### 2.9. VOC Analysis

Frozen samples of semen were thawed at room temperature, and subsequently, the vials were immersed in a water bath onto a magnetic stirrer hotplate at 60 °C overnight.

After this incubation, a Carboxen^®^/Polydimethylsiloxane (CAR/PDMS) fiber (57318, Supelco) was exposed to headspace for 15 min, as reported in [32]. GC-MS analysis of extracted volatiles was performed using GC (6890N series Agilent Technologies) coupled to MS (5973 series Agilent Technologies) equipped with a ZB-624 capillary column (Phenomenex); the injector temperature was set at 250 °C to allow thermal desorption of VOCs. The carrier gas was high-purity helium with a flow rate of 1 mL min^−1^. The MS analyses were carried out in full-scan mode with a scan range of 30–500 amu at 3.2 scans/s. Chromatograms were analyzed by Enhanced Data Analysis (MSD Chemstation E.02.02, Agilent Technologies, Santa Clara, CA, USA), and the identification of the volatile compounds was achieved by comparing mass spectra with those of the data system library (NIST14, *p* > 60%) and confirmed by the injection of external standards corresponding to most recurrent compounds. To quantify the identified VOCs, a semiquantitative method based on the internal standard (I.S.) 1,4-Dichlorobenzene-D4 (EPA-8260C) was followed. A frequency analysis was performed to identify the most frequently present VOCs.

### 2.10. Spermatozoa Staining

The Diff-Quik staining method was chosen because it better detects any abnormalities in sperm morphology [41]. The Diff-Quik staining test allows the assessment of human sperm chromatin status together with the assessment of sperm morphology as per WHO 2010. After evaluation of the fresh spermiogram, spermatozoa are fixed on the slide, stained by the Diff-Quik method, immersed in water for 1 min, and analyzed with NIKON Eclipse E100 optical MICROSCOPE, 10×, 20×, and 40× objectives, Plan E100X/1.25 Oil LENS, and DF-Fi3 color camera with phase contrast and BF brightfield. We counted at least 200 spermatozoa per sample in terms of normal/abnormal morphology and specified in the results the abnormalities found in terms of percentage in the VSR.

### 2.11. Statistical Analysis

The statistical analysis performed was the unpaired t-test for comparison of concentration and total motility between the control group and VSR group. GraphPad Prism 9.4.1 (681) was utilized for statistical analysis.

## 3. Results

### 3.1. Qualitative and Quantitative Analysis of VOCs

**Figure 2:**
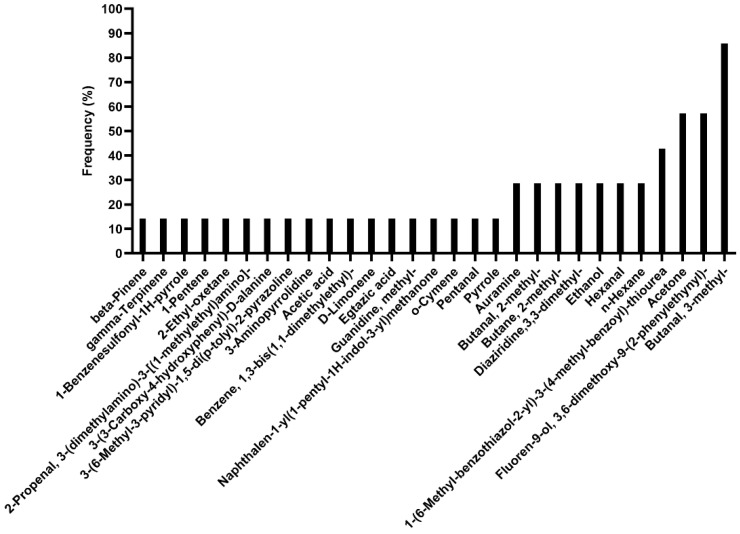
Frequency of VOCs which were detected in semen from the VSR group.

VOC analysis in sperm samples of young males from the VSR emphasized a very high inter-individual variability. As shown in Figure 2, the most detected VOC was 3-methyl-butanal, followed by acetone and fluoren-9-ol,3,6-dimethoxy-9-2-phenylethynyl-. Several VOCs were present only in 15% of samples.

### 3.2. Anthropometric Data of Individuals and Semen Analyses

**Figure 3:**
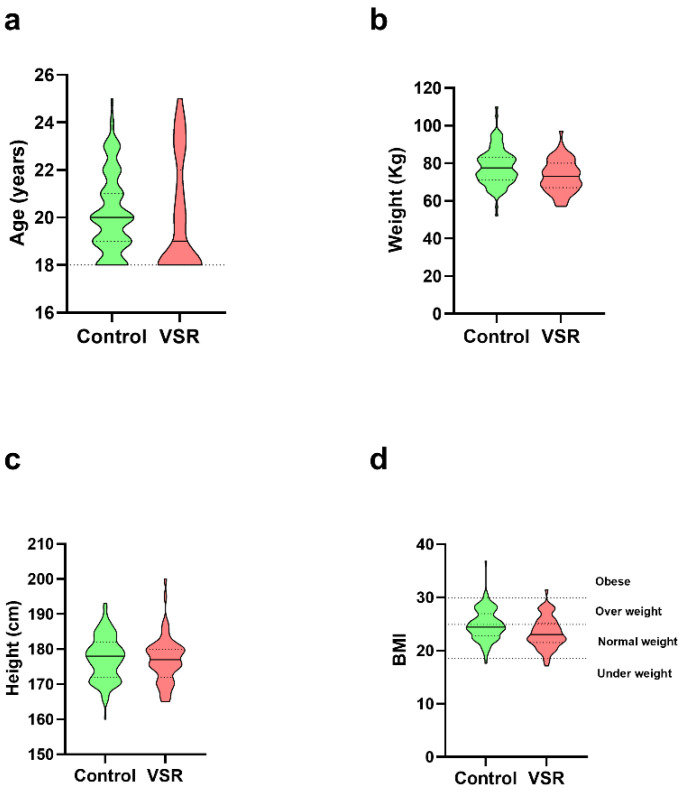
Anthropometric data: (**a**) age; (**b)** weight; (**c**) height and (**d**) BMI of control group (green) and VSR (red) individuals. BMI: Body Mass Index.

The anthropometric data of the VSR (red) displayed a very homogeneous class of subjects in terms of age (a), weight (b), height (c), and body mass index (BMI) (d) (Figure 3).

### 3.3. Semen Analysis

**Figure 4:**
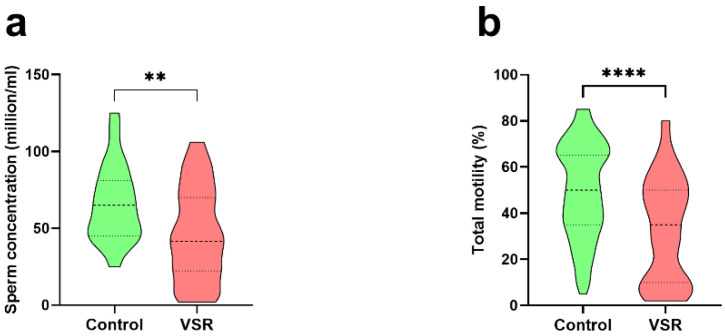
Statistical analysis of sperm concentration (**a**) and total motility (**b**) of the two groups. In green, control group, in red, VSR group. **: *p* ≤ 0.01; ****: *p* ≤ 0.0001.

The results of sperm concentration and total mobility of the semen samples are shown in Figure 4. Semen analysis indicated a statistically significant reduction in sperm concentration (66.35 ± 25.10 vs. 46.03 ± 30.23) and in total sperm motility (49.79 ± 20.41 vs. 33.09 ± 22.58) in healthy young men living in the VSR. In fact, the VSR samples showed a lower total motility than the control samples. Other seminal parameters showed significant differences, in particular immotility, non-progressive motility, and round cells (Supplementary Figure S1).

### 3.4. Evaluation of Sperm Morphology of “Valle del Sacco” Samples

**Figure 5:**
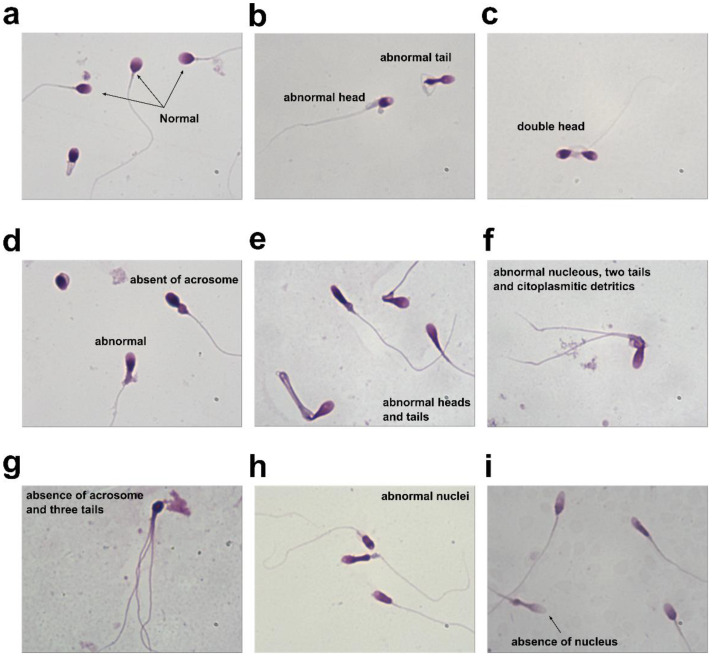
Diff-quick staining of VSR group’s spermatozoa. (**a**): control group; (**b**–**i**): VSR group.

Morphological analysis of the VSR group’s spermatozoa was performed by Diff-Quick staining. Figure 5 highlights the significant abnormalities seen in the VSR group’s spermatozoa. In particular, while the control samples showed a clear acrosome and a dark nucleus with no morphological alterations (Figure 5a), the VSR group’s spermatozoa showed a variety of different morphological alterations. They included abnormal tails and heads (Figure 5b); abnormal nuclei or absence of nuclei (Figure 5e,f,h,i); spermatozoa with a double head or double tail (Figure 5c,f) and cytoplasmatic detritus (Figure 5f); and absence of acrosomes and the presence of three tails (Figure 5d,g). In brief, about 90% of the VSR samples had various morphological changes, quantified by the sperm deformity index (SDI), such as a greater load on the head (about 40%) with altered acrosomes, pinheads, and pyriform and intermediate tract heads (about 20%) and the presence of angulations in about 10% on the tail, evaluated under the microscope by the operator and re-evaluated by photographic observation. This analysis ultimately underlined the significant alterations in spermatozoa morphology in individuals from the VSR group.

### 3.5. Analysis of SNBPs

**Figure 6:**
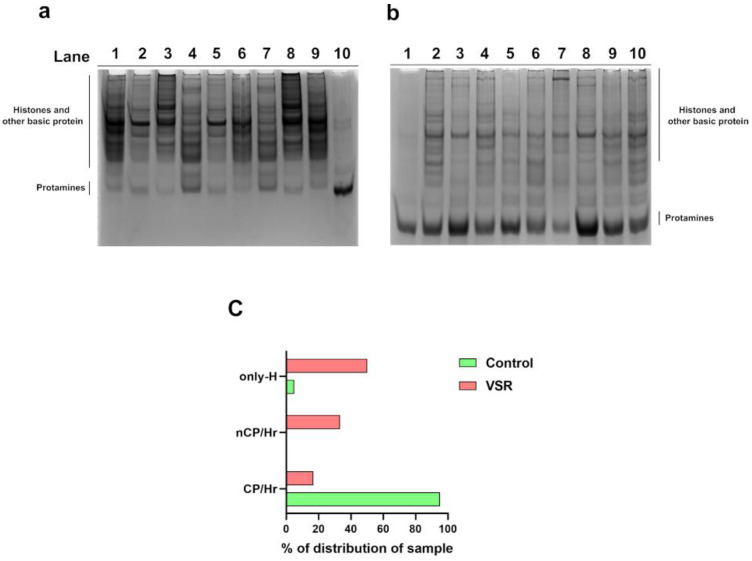
SNBP content as characterized by AU-PAGE displayed three different expression patterns. A normal canonical protamine/histone ratio (CP/Hr) was seen mainly in control group samples ((**a**), lane 10; (**b**), lane 1), while VSR samples more commonly displayed a histone-only ratio ((**a**), lanes 1–9) or a non-canonical protamine/histone ratio ((**b**), lanes 2–10). (**c**) Percentage distribution of protamines/histones ratios found in spermatozoa. In green, control group, in red, VSR group. CP/Hr: canonical protamine/histones ratio; nCP/Hr: non-canonical protamine/histones ratio; only-H: only histones.

SNBPs from samples belonging to males living in the VSR group and in the control group areas were extracted, and their protein content was characterized by acetic acid-urea gel electrophoresis (AU-PAGE) (Figure 6). The analysis by AU-PAGE showed remarkable differences between the SNBPs contained in the samples of individuals living in the two areas (Figure 6). In lane 10 of Figure 6a and in lane 1 of Figure 6b, representative electrophoretic patterns of samples belonging to the control group are shown. These samples exhibited the classic electrophoretic pattern of human SNBPs, with the canonical protamine/histone ratio (CP/Hr), which was previously reported [42]. SNBP content in the VSR samples differed from that seen in control samples and could be grouped into two main expression patterns: the first characterized by an almost complete absence of protamines and a persistence of histones (only-H) (Figure 6a, lanes 1 to 9); the second characterized by the presence of protamines and histones but in a non-canonical ratio (nCP/Hr) (Figure 6b, lanes 2 to 10). Both conditions were extremely heterogeneous (Figure 6a,b).

We classified the samples (*n* = 76) analyzed in the two areas according to the type of bands identified on AU-PAGE as specified in materials and methods. The three types of electrophoretic profiles (CP/H ratio; nCP/H ratio; and only-H) were differently distributed in the VSR and in the Valley of Sele River (control group) areas. Specifically, in males belonging to the control group, we found only two conditions, the first represented by 95.06% of the samples displaying the CP/Hr profile, and the other represented by 4.94% of the samples showing the only-H pattern (Figure 6c). In contrast, in the VSR samples, we found the CP/Hr profile only in about 16% of the individuals and only-H profile in the majority of this group’s samples (52%), while the remaining 32% of samples displayed a non-canonical protamine/histone ratio (nCP/Hr).

### 3.6. DNA Binding Ability of SNBPs Analyzed by EMSA

**Figure 7:**
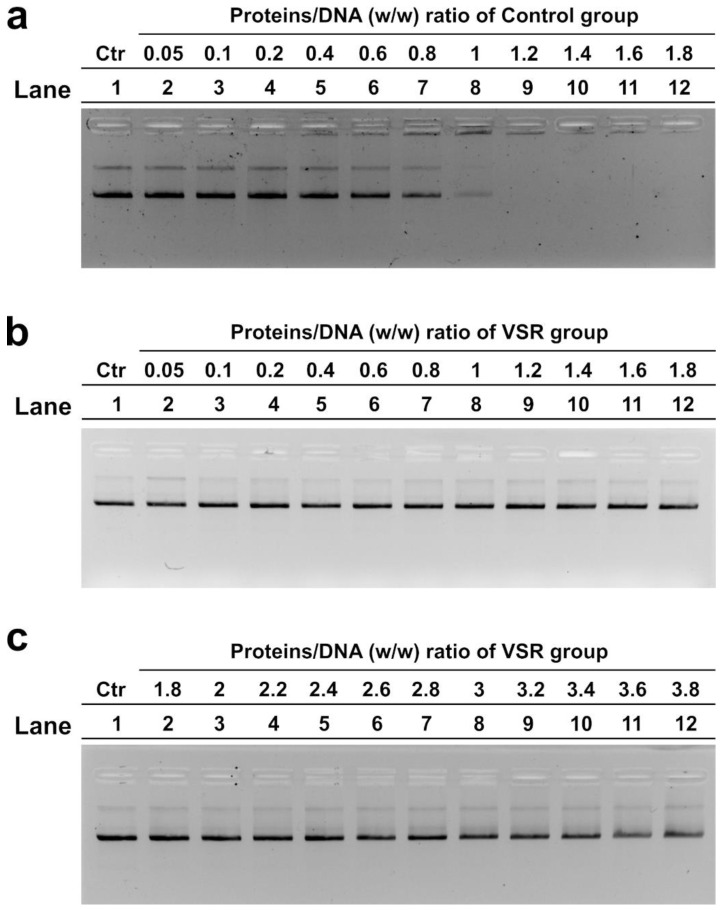
DNA-binding ability of SNBPs obtained from control group (**a**) and VSR group (**b**,**c**) analyzed by electrophoretic mobility shift assay (EMSA) on 1% agarose gel. Bands on gel representing the state of pGEM3 plasmid DNA incubated in a ratio *w/w* with increasing amount of SNBPs from samples. VSR: Valley of Sacco River; control: Valley of Sele River.

We analyzed, by electrophoretic mobility shift assay (EMSA), the differences in the DNA-binding ability of the types of SNBPs obtained in the control group and in the VSR samples. Specifically, we assessed the protein/DNA ratio required to achieve DNA saturation, which was indicated by the formation of a high-molecular-weight DNA band near the well in the electrophoretic gel as already reported [8]. The results indicated a very different DNA-binding capacity of SNBPs from the two sample groups. In fact, the SNBPs of control group samples, presenting the canonical protamines/histones ratio, produced DNA saturation at about a 1.2 *w/w* protein/DNA ratio as shown in Figure 7a, lane 9. In contrast, the SNBPs of the samples belonging to the VSR, regardless of their composition of protamines and histones, showed low DNA-binding ability because DNA saturation did not occur even at a 3.8 protein/DNA ratio as shown in the representative gel indicated in Figure 7c, lane 12. In fact, panels b and c of Figure 7 show the gels of one VSR sample, but all 76 samples of the VSR group were analyzed, and this condition was found in all VSR samples, regardless of the distribution of the protamine/histone ratio.

## 4. Discussion

Human sperm was proven in a recent study to be an excellent bioaccumulator of volatile organic compounds (VOCs) [43], and in a very recent paper of our research group, for the first time, the VOC composition of the semen of young men living in the VSR area was evaluated [32]. That work compared pollutants present in various matrices (semen, blood, etc.) in different areas of high environmental impact and showed that several VOCs were particularly present at high levels in the semen of young men living in the VSR area [32]. In particular, eight compounds presented a higher concentration in human semen samples of subjects living in the VSR with respect to another polluted area of Italy (the Land of Fires in Campania) [32]. These compounds were: 1-6-Methyl-benzothiazol-2-yl3-4-methyl-benzoyl-thiourea, 2-Methylbutane, Auramine, 3,6-Dimethoxy-9-2-phenylethynyl-fluoren-9-ol, Pyrrole, Acetic acid, D-Limonene, and 3-Aminopyrrolidinein [32]. The same VSR semen samples were used in the present work to assess whether young men living in the VSR had alterations in SNBP properties. In young men living in the VSR, we found alterations in the classical seminal parameters, such as a decreased number of spermatozoa and a lower total motility, in comparison with young men living in low environmental impact areas. In addition, by molecular approaches, we highlighted changes in the state and properties of SNBPs of young men living in the VSR area. In particular, we found an altered protamine/histone ratio in the majority of the individuals. In fact, only 16% of VSR individuals showed a normal CP/Hr, while 52% showed only-H expression and 32% showed nCP/Hr.

These alterations in the protamine/histone ratio prompted us to investigate the ability of the SNBPs to bind DNA. This was because the correct protamine/histone ratio is a prerequisite for the fertilization capacity of spermatozoa [31], and also on account of our previous work on the SNBPs of young people living in the Land of Fires, which showed that an altered protamine/histone ratio produced low DNA-binding affinity [5,7]. As a matter of fact, we found, by EMSA, that the SNBP samples of all individuals living in the VSR, regardless of their SNBP composition, showed a very low DNA-binding affinity. In fact, consistent with our previous work, it was confirmed by EMSA that the extracts of sperm nuclear basic proteins from the control group presenting a canonical protamine/histone ratio interacted with DNA in an “all or nothing” mode [44], as sperm nuclear basic protein [45] and DNA saturation, i.e., the condition in which all DNA is close to the well of agarose gel, was reached at a 1.2 protein/DNA ratio. Instead, with all samples from the VSR group, DNA saturation was never reached even at a 3.8 protein/DNA ratio. The most striking finding was the severe morphological changes observed in the VSR group’s spermatozoa. In more than 90% of the cases, severe morphological alterations were observed that were neither found in the control group nor in those from the Land of the Fires. These included abnormal tails and heads; abnormal nuclei and absence of nuclei; spermatozoa with a double head or double tail and cytoplasmic debris; absence of acrosomes and the presence of three tails. We tried to correlate the seminal and morphological alterations and the changes in the properties of SNBPs of the VSR group observed with the types of pollutants present in their semen, since it was recently demonstrated that human semen is an excellent bioaccumulator of VOCs [43]. It is known that many substances can cross the blood–testicular barrier and be released into the semen. This can lead to alterations in semen quality and quantity [46]. In particular, the VOCs that are most capable of crossing the blood–testicular barrier, or that result from biotransformations occurring at this anatomical site, are aldehydes, ethers, benzene derivatives, and terpenes [43]. As already mentioned, the main VOCs found in the semen of VSR individuals were: 1-(6-Methyl-benzothiazol-2-yl)-3-(4-methyl-benzoyl)-thiourea; Butane, 2-methyl-; Auramine; Fluoren-9-ol, 3,6-dimethoxy-9-2-phenylethynyl-; Pyrrole; Acetic acid, sodium salt; D-Limonene; and 3-Aminopyrrolidine [32]. The presence of these VOCs could be related to the poor semen quality found in the VSR cohort (Figure 4). The sperm concentration was higher than the reference value (>15 millions/mL) for most individuals, but these data have to be joined to the alarming occurrence of spermatozoa defects. The total motility was under the threshold value of 40% for a large part of the sample population [34].

The extremely toxic benzene derivatives that likely result from air pollution [47] are particularly present in semen as demonstrated by Longo et al., 2021 [32]. Benzene is a monocyclic aromatic hydrocarbon. It is a natural constituent of petroleum but is also synthesized from the other chemicals in the same petroleum. It is distributed in all biological fluids but seems to be particularly present in semen. Benzene and its derivatives can be highly dangerous because sperm cells, and consequently their DNA, are in close contact with these compounds, which in some cases also have mutagenic activity [48]. In the literature, it is reported that exposure to benzene is not only associated with the onset of aneuploidy for X, Y, and 21 chromosomes in sperm [49] but also with structural aberrations of chromosome 1 in sperm cells [50]. In addition, benzene metabolite 1,2,4-benzenetriol is able to change DNA methylation and histone acetylation of specific genes [51]. It is known that these histone modifications are crucial for the transition from histones to protamines during spermatogenesis. Thus, that benzene and its derivatives can influence these histone modifications could explain the abnormal distribution in CP/H, nCP/H, and only-H that we observed in the VSR group individuals, and in particular, the finding that the majority of subjects tested in this area presented only histones. In addition, it was suggested that histones might be targets for attack by benzene or its metabolites [52]. Since histones are central to chromatin structure and function, molecular damage to histone proteins may be able to produce the cellular changes observed in benzene toxicity. Similarly, in spermatozoa, benzene may target protamines, since the cation–π interaction made by arginine residues with aromatic ligands is more robust to changes in the environment. This interaction is the most frequently found empirically and is also calculated to be stronger than for lysine in higher-polarity environments. There are relatively few cation–π interactions involving positively charged histidine residues, although the stacked π + −π interaction is predicted to be of similar magnitude to that of arginine [53].

Aldehydes are organic compounds that carry in their structure the formyl functional group. In general, the levels of aldehydes can be considered as good markers to evaluate the levels of oxidative stress. Regarding the interactions between these compounds and human semen, the aldehyde of greatest concern is hexanal. Hexanal, mainly produced by the oxidation of linoleic acid, is used in the perfume industry to produce fruity fragrances. It is known that this compound, although it has no spermicidal activity, can significantly decrease sperm motility [54]. Therefore, the high percentage of immobile spermatozoa of these young men (about 70%) confirm the danger of hexanal in human semen.

Another potentially very toxic substance is auramine. This is used as a dye in the production of a large number of different items, such as paints, oils, and waxes. Several authors have indeed described the occurrence of dyes in the waters and sediments of rivers located within the area of influence of textile industry discharges [55,56], and this could explain the massive presence of this VOC in the semen of VSR individuals. Auramine is also used as an antiseptic and fungicide [41,42]. Auramine is known to affect DNA. In fact, this compound was shown to induce DNA fragmentation in the kidney, liver, and urinary bladder in rats [57]. Auramine is a diarylmethane dye, and the possibility to form complexes between p-sulfonatocalix[n]arenes and the amino acids lysine and arginine in water was demonstrated [58]. Therefore, it cannot be ruled out that these pollutants may bind to protamines via arginines and lead to the effects we demonstrated in the work.

Another compound that appears to significantly affect human semen is n-hexane. This alkane is used primarily as a fuel. It is an extremely harmful compound to the environment and very toxic to the reproductive system. In fact, even concentrations of 20 mg/mL induce irreversible immobility of the spermatozoa after 20 s. In addition, n-hexane can cause injury to the sperm plasma membrane [59] and may cause severe testicular atrophy and cessation of sperm production [60]. These effects could be responsible for the lower number of spermatozoa and the reduced mobility observed in VSR subjects.

Longo et al., 2021 [32] observed the preference of aldehyde, 3-methylbutanal, and 2-methylbutanal in the semen of these young men living in the VSR. Aldehydes may result in decreased sperm motility [61]. The decrease in motility could also be due to the epididymal dysfunction or elevated oxidative stress in the testicular environment [62].

As reported in a recent paper [63], pyrrole is another main VOC found in human semen, and in the study of Longo et al., 2021 [32], this substance was particularly present in subjects living in the VSR area [32]. Pyrrole is a flavoring agent with potential anti-inflammatory and antimicrobial properties [64]. Furthermore, it is the precursor of a wide variety of compounds that are known as pyrrole derivatives. 3-Aminopyrrolidine is another basis for a wide range of substances (3-aminopyrrolidine derivatives) of high pharmaceutical interest. There are also many pesticides and insecticides based on pyrrole. In the literature, it is reported that pyrroles correlate directly with the extent of testicular injury. In addition, toxicity mediated by pyrrole–protein adducts was reported [65], and pyrrole–protein adducts, pyrrole–DNA adducts, and cross-linking adducts were demonstrated [65]. Pyrrole–amino acid adducts, including pyrrole–cysteine and pyrrolelysine, were synthesized by the reaction of pyrrolic metabolites with the corresponding amino acids [66]. Very recently, Fu and co-workers reported that several pyrrole–amino acid adducts, including 7-cysteine-DHP, were identified as secondary pyrrolic metabolites that could bind to DNA, leading to the formation of pyrrole–DNA adducts [67]. Among the hypothesized mechanisms for protein cytotoxicity induced by protein adducts with pyrrole is the covalent modification of proteins with consequent impairment of the normal function of target proteins [68,69].

Additionally, ethanol consumption is one of the leading causes of male infertility; however, the mechanisms that cause ethanol-induced infertility remain unclear. Studies have shown that ethanol can cause increased frequency of abnormal spermatozoa and a reduction in mean progression rate [70], complete loss of acrosomes and loss of the equatorial segment [71], and marked decreases in testosterone levels and expression of the testicular proteins AR and TyrPho [72].

Given the particularly high concentrations of VOCs found in human semen from healthy young men from the Valley of Sacco River [32], it is presumable to suppose that these contaminants could produce the morphological- and molecular-level alterations in the spermatozoa of these subjects. Indeed, both the significant reduction in motility and the high incidence of morphological abnormalities in sperm samples from the Valley of Sacco River are worrying indicators indicating a strong influence of exposure to highly contaminated living environments. Finally, our molecular biology-based approach confirmed the presence of critical alterations in SNBP properties. It is well-known that proper chromatin compaction of spermatozoa is critical for the fertilizing capacity of these cells. Of course, the alterations observed at the level of protamine/histone ratios and DNA-binding affinity of these proteins suggested that there was improper chromatin compaction in these spermatozoa, and thus, that there have been disturbances in the processes that occur at the end of spermatogenesis. The canonical protamine/histone ratio is crucial for the formation of a correct sperm chromatin structure, which is the prerequisite for the success of fertilization. Indeed, the high content of arginine residues in protamines allows them to bind to both the minor and major grooves of DNA, resulting in an appropriate compactness of the sperm chromatin, whereas histones only interact with a specific region of sperm DNA, producing less compact chromatin [45]. Considering that 10–15% of histones are maintained in the chromatin of human sperm [73,74,75,76], it may be assumed that the presence of protamines and histones in altered ratios may determine not only an unstable binding to DNA but also a decreased protection of DNA from external agents, such as various pollutants. Furthermore, in samples containing only histones, the low degree of sperm chromatin compactness could cause greater exposure of the DNA to external interferents. Indeed, it has been shown that some VOCs can cause a significant increase in histone deacetylation. This effect may be particularly significant in the histone–protamine transition, which requires histone acetylase activity, and could therefore be the reason for the high percentage of subjects (around 52%) in VSR areas who had only histones in their spermatozoa.

Given the varied negative effects of VOCs on spermatozoa, it is possible to speculate that their presence in the semen of these subjects may lead to synergistic negative effects on the functionality of these cells. Of course, this work has some limitations because we did not conduct VOC analysis on semen samples from subjects residing in the Valley of Sele River. Therefore, we can only speculate that the drastic changes observed at the morphological and molecular levels in the spermatozoa may be due to VOCs, since VOCs were found to accumulate in the sperm of these young men. As already mentioned, the Sele Valley, on the basis of the pollutant concentrations detected in the area, is included in the ‘maintenance’ category and is classified as a low diffuse emission area for sulfur oxide and as a medium diffuse emission area for nitrogen oxide, carbon monoxide, volatile organic compounds, and suspended particulate matter, for which the human health risk thresholds are not exceeded [33]. Therefore, it can be supposed that since there are no excesses of volatile organic compounds in this area, high levels of VOCs should not be found in the semen of its residents. In conclusion, our findings represent preliminary data that need to be further investigated, but they nevertheless indicate great concern for the residents of that area. This is particularly noticeable taking into account that the subjects recruited in the VSR area are very young and healthy men in the prime of their fertile life. They were recruited randomly but met the selection criteria described. They are subjects who had never had a seminal examination. The context of alleged fertility problems derives from the fact that the VSR area is a site of national interest; in fact, several studies in Italy have reported that in areas of high environmental pressure, there is an increase in infertility, urogenital malformations, and chronic diseases (cancer, diabetes, etc.) [77]. Although the subject of infertility is mentioned in the text as an alarming fact in the population living in the area, we cannot absolutely state that our volunteers living in the VSR are infertile, but we simply state that their seminal quality presents alarming characteristics in terms of morphology, protamine: histone ratio, and seminal parameters. Such characteristics are crucial to understand why a couple is infertile or to suggest the couple to follow an assisted fertilization procedure [1,2,78]. For this reason, as future goals, we propose to measure the levels and types of VOCs in the young men of the Valley of Sele River of this sampling and to look for more precise correlations between particular VOCs found in the semen of the young men of the Sacco Valley and morphological and molecular alterations in the spermatozoa. An aliquot of the ejaculate was not foreseen for the VOC analysis at the time of the recruitment and collection of samples, as the authors responsible for this analysis joined the EcoFoodFertility initiative later on. However, such comparative analysis is planned to be performed in future recruitment and human biomonitoring studies in those areas. The Sele Valley is an area where VOCs are not detected in the environment at the levels found in the Sacco Valley, so presumably, they should not be present in the seminal fluid, but in any case, we will check for their presence in the next planned study. Campania, compared to Lazio, also does not have a high prevalence of VOCs, as shown by the comparison between the Land of Fires and Sacco Valley [32].

The consequences on the future reproductive potential of young people living in the VSR area therefore cannot be underestimated, especially given that semen represents an excellent marker of environmental and general health [79,80,81,82].

## 5. Conclusions

The limitations of this study lie in the fact that we did not carry out analyses of VOCs in the semen samples of subjects living in the Sele Valley. The fact remains, however, that despite the limitations of our study, our results showed very serious seminal alterations in these subjects, which have never been observed in other areas characterized by other types of pollutants. We therefore believe that there is a high probability that the chemical exposure of the population living in the Sacco River Valley has harmful effects on male infertility. Although our volunteer residents in the VSR cannot be defined as strictly infertile, the quality of their sperm presents alarming characteristics in terms of morphology, protamine/histone ratio, and seminal parameters. This is very relevant considering that the volunteers were very young men (18–20 years old) who should be in full reproductive capacity. These characteristics are also crucial to understand why a couple is infertile or to suggest the couple to follow an assisted fertilization procedure.

## Supplementary Materials

The following supporting information can be downloaded at: https://www.mdpi.com/article/10.3390/ijerph191711023/s1, Figure S1: The figure shows the seminal parameters of two groups. Green: control group; red: VSR group. Unpaired T-test performed with GraphPad Prim ver. 9.4.1 (681). ns: *p* > 0.05; é; *: *p* ≤ 0.05; **: *p* ≤ 0.01; ***: *p* ≤ 0.001; ****: *p* ≤ 0.0001.

## Abbreviations

VSR	Valley Of Sacco River
EMSA	Electrophoretic Mobility Shift Assays
EDTA	Ethylenediaminetetraacetic Acid
VOCs	Volatile Organic Compounds
SNBP	Sperm Nuclear Basic Protein
CP/Hr	Canonical Protamines/Histones Ratio
nCP/Hr	Not Canonical Protamines/Histones Ratio
Only-H	Only histones and other basic proteins
BMI	Body Mass Index
AU-PAGE	Acetic Acid-Urea Gel Electrophoresis
PMSF	Phenylmethylsulfonyl Fluoride
DTT	Dithiothreitol
TCA	Trichloro Acetic Acid
TBE	Tris-Borate-EDTA
