# Supplementary material for: Molecular Alterations and Severe Abnormalities in Spermatozoa of Young Men Living in the “Valley of Sacco River” (Latium, Italy): A Preliminary Study
Source: Int J Environ Res Public Health. 2022 Sep 3;19(17):11023. doi: 10.3390/ijerph191711023 (PMC9518305; doi:10.3390/ijerph191711023)
Supplement: Supplementary file 1 [file ijerph-19-11023-s001.zip › ijerph-1839349-supplementary.pdf]

Molecular Alterations and Severe Abnormalities in Spermatozoa of Young Men Living in the "Valley of Sacco River" (Latium, Italy): A Preliminary Study

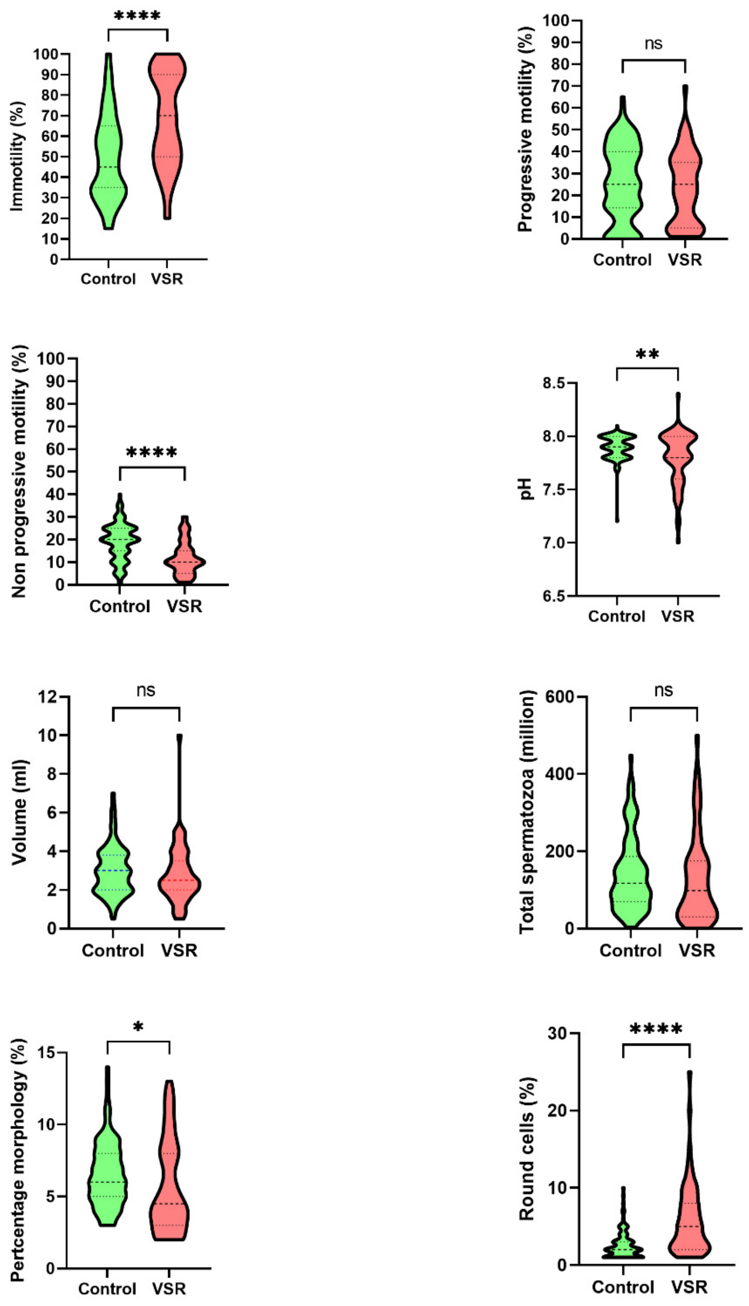

Figure S1. The figure shows the seminal parameters of two groups. Green: control group; red: VSR group. Unpaired T-test performed with GraphPad Prim ver. 9.4.1 (681). ns: $p > 0.05$; é; *: $p \leq 0.05$; **: $p \leq 0.01$; ****: $p \leq 0.0001$.
